# Zinc intake ameliorates intestinal morphology and oxidative stress of broiler chickens under heat stress

**DOI:** 10.3389/fimmu.2023.1308907

**Published:** 2024-01-08

**Authors:** Ping Hu, Kaiqi Li, Xiaoxu Peng, Tongjia Yao, Chuyang Zhu, Haotian Gu, Hao-Yu Liu, Ming-an Sun, Yun Hu, Wael Ennab, Xugang Luo, Demin Cai

**Affiliations:** ^1^ College of Animal Science and Technology, Yangzhou University, Yangzhou, China; ^2^ International Joint Research Laboratory in Universities of Jiangsu Province of China for Domestic Animal Germplasm Resources and Genetic Improvement, Yangzhou University, Yangzhou, China; ^3^ Institute of Comparative Medicine, College of Veterinary Medicine, Yangzhou University, Yangzhou, China; ^4^ Department of Veterinary Biomedical Sciences, Botswana University of Agriculture and Agriculture and Natural Resources, Gaborone, Botswana

**Keywords:** zinc, heat stress, oxidative stress, intestinal morphology, broiler

## Abstract

Zinc (Zn), an essential trace element for poultry, plays a crucial role in promoting growth, improving feed conversion efficiency, enhancing antioxidant activity, and preventing disease. This study investigated the impact of different levels and sources of dietary Zn supplementation on the growth performance, intestinal morphology and antioxidant activity of broiler chickens under heat stress conditions. In this experiment, 1024 Xueshan chickens were divided into eight groups and subjected to heat stress conditions with different levels of Zn supplementation (30 mg/kg, 60 mg/kg, and 90 mg/kg) using organic or inorganic sources. Our findings indicated that dietary Zn supplementation significantly increased the feed-to-weight ratio of broilers during the experimental period under heat stress. Moreover, Zn supplementation positively increased the villus height and villus width in the jejunum and ileum at 74 and 88 days old, with the 60 and 90 mg/kg groups outperforming other groups, and organic Zn was more effective than inorganic Zn. Furthermore, Zn supplementation significantly increased serum antioxidant levels, with higher superoxide dismutase (SOD), malondialdehyde (MDA), and glutathione peroxidase (GSH-px) activities, and organic Zn was more effective than inorganic Zn. This study concludes that Zn supplementation is beneficial in mitigating the detrimental impacts of heat stress on broilers. The findings suggest that employing Zn as a strategy can enhance productivity in the poultry industry by positively influencing intestinal morphology and bolstering antioxidant activity to counteract potential stress.

## 1 Introduction

Zinc (Zn) is an important trace element widely found in poultry and is mainly located in the liver and bones. It is one of the essential trace elements for the growth and proper functioning of the immune system in poultry. Zinc can promote the poultry’s growth and development, improve the feed conversion rate, enhance their immunity, and prevent the occurrence of diseases (1). Therefore, it is important to add the right amount of zinc to poultry feed to help farmers improve poultry production and quality. Studies have shown that zinc deficiency can lead to growth retardation, reduced immunity, and susceptibility to disease in poultry (2–4). For animals, zinc is exclusively sourced from the external environment, making self-synthesis impractical. Consequently, incorporating zinc into the feed emerges as a crucial measure to ensure the healthy growth of poultry (5).

Broiler chickens need to grow rapidly, therefore the amount of zinc in their bodies is particularly important. The National Research Council (NRC) recommends 40 mg/kg of zinc in the feed ration to maintain the optimal production performance of poultry (6). Zinc is mainly classified into organic and inorganic zinc. In the poultry production, two common zinc sources are used, inorganic zinc and organic zinc, while organic zinc has been reported to be more effective than inorganic zinc in increasing growth rate, improving feed conversion, reducing antagonistic reactions with other minerals in the feed, and reducing raw material wastage and farm pollution (6–8).

Heat stress occurs when the body is exposed to high temperatures or a combination of high temperature and humidity that overwhelms the body’s ability to dissipate heat (9). Heat stress can lead to responses in the animal’s body such as panting, increased heart rate, water and electrolyte imbalance, and an increase in oxidative metabolism and peroxides in the body (10, 11). It also results in damage to the membrane system and an acceleration of thyroid and adrenal functions, endangering the life of animals (12, 13).

Modern livestock farming often seeks to maximize profits by raising animals with rapid growth rates. Broiler chickens, in particular, are often raised in high-density conditions, and these fast-growing animals are typically sensitive to environmental changes and have poor adaptability (14). Under heat stress conditions, the gastrointestinal tract experiences ischemia and hypoxia early on and recovers last, making it susceptible to damage or failure at an early stage (15). This significantly reduces the feed intake and growth rate of broiler chickens, lowers their egg production performance, and reduces their reproductive and hatching capabilities (16).

Currently, zinc is used as a dietary supplement for poultry to prevent oxidative damage and intestinal injury caused by heat stress (17). Zinc can reduce the harmful effects of heat stress possibly by increasing CuZn-SOD activity (18), and increased MT concentration (19). Zinc can effectively improve animal gut health, and promote the digestion, absorption, and utilization of nutrients from feed (20). Organic zinc is most effective in ameliorating heat stress, which can improve broiler performance to some extent, increase the antioxidant capacity of broilers, significantly increase the length of small intestinal villi, and decrease the depth of crypt foci in piglets (21, 22).

Moreover, zinc enhances antioxidant capacity in poultry by increasing the activity of copper-zinc superoxide dismutase and zinc metalloenzymes and by altering DNA and chromatin structure to influence gene expression (3, 23). However, the enzyme activity is greatly affected by temperature, and when the ambient temperature is too high, it significantly alters the activity of metabolic enzymes in the body, hence, increasing the metabolic rate and thereby increasing the production of free radicals. Excessive free radicals disrupt the body’s oxidative and antioxidative balance, leading to lipid peroxidation, DNA and protein damage, and the generation of oxidative stress (24, 25).

So far, the evaluation of zinc levels in broiler chickens under heat stress conditions has mainly focused on growth performance, reproductive performance, meat quality, serum, and tissue zinc content, and so on (26–28). However, most research focuses on the impact of different sources or levels of zinc on intestinal function in broiler chickens under heat stress, with few studies exploring the combined effects of different zinc sources and levels on intestinal function in broiler chickens under heat stress (29, 30). Therefore, the aim of this experiment is to investigate the effects of different levels of organic or inorganic zinc in the diet on growth performance, small intestinal morphology and antioxidant function in broiler chickens under heat stress. The specific sensitive indicators will be selected in this experiment, and the best statistical model will be fitted to accurately assess the zinc requirements in corn-soybean meal-based diets for broiler chickens aged 61-102 days under heat stress. This will provide experimental evidence for the scientific and rational zinc supplementation in the production of broiler chickens under heat-stress conditions.

## 2 Materials and methods

### 2.1 Ethics statement

The animal research proposal was approved by the Institutional Animal Care and Utilization Committee (IACUC) of the Animal Experimental Ethics Committee of Yangzhou University (Permit Number: SYXK (SU) IACUC 2012-0029).

### 2.2 Experimental design and treatments

This experiment was conducted using 1024 healthy commercial Xueshan female chickens (purchased from Jiangsu Lihua Farming Co., Ltd.), 50 days old with the same body weight, which have been meticulously developed through the crossbreeding of high-quality local Tibetan chickens and Chahua chickens. From 50 to 60 days of age, all chickens were uniformly fed ad libitum with the same corn-soybean meal-type full-price ration (purchased directly from Jiangsu Lihua Farming Co., Ltd.). At 61 days of age, the chickens were divided into 8 experimental groups and each group was divided into 8 replicates with 16 chickens in each replicate (consisting of 2 adjacent cages with 8 chickens in each cage). The cages were stainless steel cages with a length × width × height of 90 × 70 × 45 cm. The experiment lasted from day 61 to day 102, spanning a total of 42 days. The factorial experimental design was used in this experiment involving a two-factor. The factors included zinc supplementations (organic or inorganic Zn) and Zinc levels (30, 60 or 90 mg/kg). The heat stress conditions were implemented from 9:00 to 17:00, maintaining a temperature of 34 ± 1°C for a duration of 8 hours per day. Outside this timeframe, the temperature was sustained at 28 ± 1°C for the remaining hour. Under both heat stress and normal temperature conditions, the relative humidity was kept at 55 ± 5%. Two different sources of zinc were added, namely inorganic zinc sulfate (ZnSO_4_.7H_2_O, Zn content calculated as 22.62%) and organic protein-bound zinc with moderate chelation strength (Organic Zn, 17.09% Zn, and Qf = 51.6 from analysis). Three levels of zinc supplementation in the diets were established based on meeting the zinc nutritional requirements for 61-102 days old Xueshan chickens (31). These levels were set at 30 mg/kg, 60 mg/kg, and 90 mg/kg of zinc. Additionally, a corn-soybean meal-based basal diet without added zinc was used as the negative control group (**Supplementary Table S1**).

Two factors completely randomized experimental design as follows and outlined in **Table 1** in the appendix (1): Negative control diet (CON) without zinc addition (2), Positive control diet with zinc sulfate addition to meet zinc requirement (Inorganic Zn-CON) (3), Zinc addition diet based on positive control diet. During the test period, constant light was provided for 24 hours per day, routine immunization was performed, and all other feeding and management of the chickens were carried out following the “Xueshan Chicken Feeding and Management Manual”. All animal experiments conducted in this study have received ethical approval from the Animal Experiment Ethics Review at Yangzhou University.

**Table 1:** Effects of different Zn sources and Zn levels on growth performance and mortality of Jiangsu Xueshan broilers of different ages under heat stress^1^.

Items	Zn level(mg/kg)	61-74 days				75-88 days				89-102 days			
		ADG (g/d)	ADF (g/d)	Feed-to gain ratio	Mortality (%)	ADG (g/d)	ADF (g/d)	Feed-to gain ratio	Mortality (%)	ADG (g/d)	ADF (g/d)	Feed-to gain ratio	Mortality (%)
CON	0	21.0	59.0^##^	2.84^#^	0	11.5	69.4	6.35	0	14.8^##^	67.4	5.22	0
Inorganic Zn- CON	50	19.8	65.0	3.37	0	11.8	69.7	5.98	0	10.6	66.6	7.22	0
Inorganic Zn^@^	30 ^&^	21.2	69.7	3.37	0	7.6	62.5	8.06	0	15.0	61.4	7.01	0
	60 ^&^	20.2	63.9	3.36	0	13.3	74.8	5.67	0	11.2	64.2	7.48	0
	90 ^&^	21.3	69.4	3.31	0	12.5	63.4	5.58	0	14.4	66.8	5.27	3.13
Organic Zn^$^	30 ^&^	20.9	63.9	3.21	0	9.1	62.2	4.01	0	15.7	66.0	4.96	0
	60 ^&^	19.8	60.5	3.09	0	13.1	66.5	5.72	0	8.4	66.5	4.71	3.13
	90 ^&^	21.7	62.9	2.94	0	10.5	79.5	8.34	0	17.0	66.7	4.11	3.13
SD		101.42	13.00	35.07	0	3.50	38.22	62.50	0	63.54	3.00	25.54	1.57
Zn source	Inorganic Zn	20.9	67.6	3.35	0	11.1	66.9	6.44	0	13.5	64.0	6.64	0.78
	Organic Zn	20.8	62.4	3.08	0	10.9	69.4	6.02	0	13.8	66.4	4.61	2.09
SD		27.50	19.50	11.50	0	43.50	4.50	23.50	0	12.50	21.00	9.00	0.66
Zn level	30	21.3	65.1	3.29	0	8.4	62.4	6.04	0	15.3	63.7	5.99	0
	60	20.0	62.2	3.23	0	13.2	70.6	5.70	0	9.8	65.3	6.18	1.57
	90	21.5	66.1	3.13	0	11.5	71.4	6.96	0	15.8	65.5	4.69	3.13
SD		1.26	7.24	0.36	0	1.97	9.00	1.58	0	4.18	4.720	1.10	1.28
*P value*	Zn source	0.946	0.201	0.260	0	0.890	0.951	0.802	0	0.947	0.350	0.134	\
	Zn level	0.590	0.602	0.856	0	0.051	0.343	0.811	0	0.035	0.591	0.626	\
	Zn source*Zn level	0.962	0.994	0.941	0	0.671	0.198	0.246	0	0.540	0.723	0.880	\

^1^All groups are subject to heat stress.

^$^Inorganic Zn: ZnSO4.7H2O.

^@^Organic Zn: Medium chelation strength organic protein Zn.

^&^Zn is added to the positive control diet.

^#^It indicates that CON group is significantly different from the single degree-of-freedom comparison performed in groups 2-8 (P<0.05). ^##^Indicates a high significant difference (P<0.01).

During the experiment, the health condition of chickens was observed and recorded every day, and if any chickens were sick or dead, they were immediately dissected, the cause of death was observed and analyzed, the test site and chicken house were clean and sanitary, and normal disinfection was carried out, the test chicken house was well ventilated, and the required temperature was maintained; the feed was produced according to the standard, and the quality of drinking water was good. At the end of every two weekends, the chickens were weighed in fasting weight, and the feed intake was counted. The average daily weight gain, feed intake, feed intake/gain ratio, and mortality rate of the chickens was calculated.

### 2.3 Sample collection and preparation

The samples of corn, soybean meal, and the diets of each treatment group were collected on-site during the preparation of the experimental diets. The samples were ground through a 200-mesh sieve and stored in a self-sealing bag at low temperature for the analysis of the crude protein and calcium content of the feed materials and the diets.

On the 74th, 98th, and 102nd days of the experiment, following the methods from previous research (32), after an overnight fasting period with access to water, two chickens from each replicate group were selected, and the blood samples were collected from the wing vein of each chicken. After being centrifuged at 3000 r/min for 10 minutes, the serum was collected and frozen at -20°C for the determination of antioxidants enzymes activity. Subsequently, the chickens were euthanized, and the small intestine was carefully removed. The jejunum and ileum segments were isolated, and sections (2 cm) from the middle of jejunum and ileum were then excised. These excised portions were preserved in a 4% paraformaldehyde solution for subsequent morphological studies. The duodenal, jejunal, and ileal mucosa were carefully scraped with a microscopic glass slide and stored at −80°C for analysis of gene expression and oxidative status (33).

### 2.4 Sample analysis

The crude protein content in feeds was determined using the Kjeldahl method (GB/T 6432-1994). After digestion of the samples with nitric acid and perchloric acid wet method, the calcium content in feed ingredients and feeds was measured using an Optima 7300 DV Inductively Coupled Plasma Optical Emission Spectrometer (Optima 7300 DV, PerkinElmer, USA).

For fixed tissues, hematoxylin and eosin staining was performed (34) followed by microscopic observation and image processing and analysis system (Version 1, Leica Imaging Systems Ltd, Cambridge, UK). Villus height (VH, μm), crypt depth (CD, μm), and villus width (VW, μm) measurements were performed from 3 different regions of each section (with intact villi and straight orientation), and the average values were taken as the measurement results (35).

Total antioxidant capacity, and the activities of various enzymes in different segments of the small intestine mucosa, including superoxide dismutase (SOD) and glutathione peroxidase (GSH-px) were planned to be determined using commercial assay kits. Additionally, the malondialdehyde (MDA) content was planned to be measured. Additionally, the malondialdehyde (MDA) content was planned to be measured. All experimental procedures were accurately performed following the manufacturer’s instructions (GSH-PX, A005; SOD, A001-1; MDA, A003-1) (Jiancheng Bioengineering Institute).

### 2.5 Data statistical analysis

The Shapiro-Wilk test was used to assess the normality distribution of the data. Statistical analysis of the experimental data was conducted using the SAS 9.4 (2013) system. In this analysis, the single-degree-of-freedom comparison method (SDOF) was used separately to test the significance of differences between the negative control group and all zinc-treated groups under heat stress conditions, as well as between the positive control group and the groups treated with 30, 60, and 90 mg/kg of zinc (36). A general linear model (GLM) procedure was applied to analyze the data under heat stress conditions for all groups except the negative and positive control groups, using a 2 × 3 two-factor analysis of variance (ANOVA) design. The statistical factors considered include the source of zinc, the level of zinc supplementation, and their interaction. For statistically significant differences observed in the ANOVA, the least significant difference (LSD) method was used to compare the significance of differences between the means. Mortality data for the chickens were subjected to arcsine transformation before statistical analysis, with each replicate treated as an experimental unit. Data were presented as the mean and standard deviation of the mean. Differences were considered to be statistically significant at (*P*< 0.05).

## 3 Results

### 3.1 Effect of dietary zinc addition on broiler chickens performance

As shown in **Tables 1** and **Supplementary Table S2**, under heat stress conditions, dietary zinc supplementation significantly enhanced the average daily feed intake and feed/weight gain ratio of broiler chickens at 74 days of age, with sustained improvements in the feed/weight gain ratio throughout the experimental period (*P*<0.05). Moreover, no significantly difference about mortality at the age of 74, 88 and 102 days was observed in all groups (*P*>0.05). Therefore, a tentative conclusion can be drawn that the inclusion of zinc in the diets improves the feed-to-weight gain ratio of broiler chickens under heat stress.

### 3.2 Effect of dietary zinc supplementation on jejunal and ileal morphology

As shown in **Table 2**, zinc supplementation exhibited a significant increase in the jejunal villus height (VH), villus width (VW), and the VH to crypt depth (CD) ratio in broiler chickens under heat stress at 74, 88, and 102 days (*P*<0.05). Notably, at 88 and 102 days, the impact of organic zinc was more pronounced on VH compared to inorganic zinc (*P*<0.05). Furthermore, at the age of 88 days, a dose of 30 mg/kg of zinc resulted in a significantly higher VH and VH to CD ratio compared to other doses (*P*<0.05).

**Table 2:** Effects of different Zn sources and Zn levels on jejunal morphology of 61–102day old Xueshan chickens under heat stress^1^.

Items	Zn level (mg/kg)	74 days				88 days				102 days			
		VH (μm)	VW (μm)	CD (μm)	VH/CD	VH (μm)	VW (μm)	CD (μm)	VH/CD	VH (μm)	VW (μm)	CD (μm)	VH/CD
CON	0	702^##^	108^##^	233	3.01^##^	922^#^	108^#^	253	3.92^#^	772^#^	151^##^	241	3.20^#^
Inorganic Zn-CON	50	1038	174	295	3.52	1215	216	313	3.88	883	157	265	3.33
Inorganic Zn^$^	30 ^&^	892	172^ab^	273	3.27	1031^b^	121	216^c^	4.77	973	166^b^	228	4.27
	60 ^&^	877	193^a^	202	4.34	1012^bc^	92	241^bc^	4.20	832	91^d^	233	3.57
	90 ^&^	963	137^bc^	223	4.32	826^d^	154	192^d^	4.30	861	133^bc^	194	4.44
Organic Zn ^@^	30 ^&^	792	138^bc^	226	3.50	1122^a^	131	252^b^	4.45	888	241^a^	224	3.96
	60 ^&^	1054	133^c^	299	3.53	1012^bc^	117	226^bc^	4.48	1011	162^b^	183	5.52
	90 ^&^	1052	112^c^	242	4.35	995^c^	92	311^a^	3.20	842	113^c^	193	4.36
SD		101.42	13.00	35.07	0.60	3.50	38.22	62.50	0.89	63.54	3.00	25.54	0.65
Zn source	Inorganic Zn	911	167	233	3.91	956^b^	122	216	4.43	889^b^	130^b^	218	4.10
	Organic Zn	966	128	256	3.77	1043^a^	113	263	3.97	914^a^	172^a^	200	4.57
SD		27.50	19.50	11.50	0.07	43.50	4.50	23.50	0.23	12.50	21.00	9.00	0.24
Zn level	30	842	155^a^	250	3.37	1077^a^	126	234	4.60^a^	931^a^	204^a^	226	4.12
	60	966	163^a^	251	3.85	1012^b^	105	234	4.32^a^	922^a^	127^b^	208	4.43
	90	1008	125^b^	233	4.33	911^c^	123	252	3.62^b^	852^b^	123^b^	194	4.39
SD		70.47	16.36	8.26	0.39	68.30	9.27	8.49	0.41	35.31	37.28	13.10	0.13
*P value*	Zn source	0.358	0.009	0.334	0.656	<0.001	0.551	0.001	0.122	0.335	<0.001	0.185	0.057
	Zn level	0.113	0.030	0.702	0.355	<0.001	0.348	0.319	0.012	0.033	<0.001	0.222	0.243
	Zn source* Zn level	0.191	0.337	0.490	0.476	0.002	0.140	<0.001	0.043	0.001	0.004	0.304	0.004

^1^All groups are subject to heat stress. Values represent the average of 1-8 replicate cages. VH, Villus height; VW, Villus width; CD, Crypt depth; VH/CD, Villus height to Crypt depth ratio.

^a-d^Values with different superscript letters in the same column differ significantly (P < 0.05).

^$^Inorganic Zn: ZnSO_4_.7H_2_O.

^@^Organic Zn: Medium chelation strength organic protein Zn.

^&^Zn is added to the positive control.

^#^It indicates that CON group is significantly different from the single degree-of-freedom comparison performed in groups 2-8 (P<0.05). ^##^Indicates a high significant difference (P<0.01).

Additionally, zinc supplementation demonstrated a positive effect on the ileal VH, VW, and VH to CD ratio in broiler chickens under heat stress at 74, 88, and 102 days (*P*<0.05) (**Table 3**). Moreover, at 74 and 102 days, the VH was significantly higher at a dosage of 90 mg/kg compared to 30 and 60 mg/kg (*P*<0.05). Furthermore, the VH to CD ratio was also higher at 90 mg/kg at the age of 102 days compared to other doses (*P*<0.05). Consequently, the addition of zinc supplementation appears to have a beneficial impact, positively alleviating and enhancing intestinal morphology under heat stress.

**Table 3:** Effects of different zinc sources and zinc levels on ileal morphology of 61–102day old Xueshan chickens under heat stress^1^.

Items	Zn level (mg/kg)	74 days				88 days				102 days			
		VH (μm)	VW (μm)	CD (μm)	VH/CD	VH (μm)	VW (μm)	CD (μm)	VH/CD	VH (μm)	VW (μm)	CD (μm)	VH/CD
CON	0	584^##^	92^#^	152	3.84^##^	858	91^##^	196^##^	4.30^##^	571^##^	77^##^	173	3.30^##^
Inorganic Zn-CON	50	872	118	194	4.49	952	91	191	4.98	846	121	196	4.32
Inorganic Zn^$^	30 ^&^	714^d^	99	155	4.61	682	158	153	4.46	755^c^	109	155	4.87^b^
	60 ^&^	623^e^	138	122	5.11	877	131	146	6.01	751^c^	62	181	4.15^c^
	90 ^&^	737^c^	112	157	4.69	752	113	124	6.06	821^b^	139	166	4.95^b^
Organic Zn^@^	30 ^&^	385^f^	124	161	2.39	926	98	167	5.54	471^d^	97	172	2.74^d^
	60 ^&^	798^b^	95	198	4.03	953	112	222	4.29	767^bc^	81	142	5.40^a^
	90 ^&^	883^a^	92	143	6.17	888	98	192	4.63	922^a^	94	168	5.49^a^
SD		94.00	13.00	23.54	0.99	90.75	21.65	28.80	0.64	137.50	23.38	15.14	0.51
Zn source	Inorganic Zn	691	116	145	4.77	770^b^	134	141^b^	5.46	776	103	167	4.65
	Organic Zn	689	104	167	4.13	922^a^	103	194^a^	4.75	720	91	161	4.47
SD		1.00	6.00	11.00	0.32	76.00	15.50	26.50	0.36	28.00	6.00	3.00	0.09
Zn level	30	550^c^	112	158	3.48	804	128	160	5.03	613^c^	103^ab^	164	3.74^b^
	60	711^b^	117	160	4.44	915	122	184	4.97	759^b^	72^b^	162	4.69^a^
	90	810^a^	102	150	5.40	820	106	158	5.19	872^a^	117^a^	167	5.22^a^
SD		107.15	6.24	4.32	0.78	48.99	9.29	11.81	0.09	106.02	18.80	2.05	0.61
*P value*	Zn source	0.953	0.085	0.205	0.160	0.002	0.031	0.001	0.038	0.352	0.433	0.691	0.871
	Zn level	0.005	0.509	0.816	0.077	0.128	0.316	0.137	0.970	0.004	0.046	0.997	0.040
	Zn source* Zn level	0.003	0.026	0.073	0.889	0.356	0.328	0.075	0.972	0.034	0.079	0.336	0.040

^1^All groups are subject to heat stress. Values represent the average of 1-8 replicate cages. VH, Villus height; VW, Villus width; CD, Crypt depth; VH/CD, Villus height to Crypt depth ratio.

^a-f^Values with different superscript letters in the same column differ significantly (P < 0.05).

^$^Inorganic Zn: ZnSO_4_.7H_2_O.

^@^Organic Zn: Medium chelation strength organic protein Zn.

^&^Zn is added to the positive control.

^#^It indicates that CON group is significantly different from the single degree-of-freedom comparison performed in groups 2-8 (P<0.05). ^##^Indicates a high significant difference (P<0.01).

### 3.3 Effect of dietary zinc supplementation on serum antioxidants activity

From the data in **Table 4**, it can be noted that zinc supplementation decreased the MDA contents and increased SOD and GSH-px enzyme activities under heat stress conditions compared with the CON group in 74 and 88 days (*P*<0.05). Additionally, the utilization of organic zinc exhibited a more pronounced effect, leading to elevated SOD and GSH-px activities and a reduction in MDA content at both 74 and 88 days, in contrast to inorganic zinc (*P*<0.05). Noteworthy variations in both zinc sources and levels were observed to exert discernible influences on SOD and GSH-px enzyme activities, as well as MDA contents. Of particular significance, GSH-px enzyme activity demonstrated a notable increase at a zinc concentration of 60 mg/kg at the age of 88 and 102 days (*P*<0.05).

**Table 4:** Effects of different zinc sources and zinc levels on serum antioxidant levels of 61–102 days old Xueshan chickens under heat stress.

Items	Zn level (mg/kg)	74 days			88 days			102 days		
		MDA (nmol/mL)	SOD (U/mL)	GSH-px (U/mL)	MDA (nmol/mL)	SOD (U/mL)	GSH-px (U/mL)	MDA (nmol/mL)	SOD (U/mL)	GSH-px (U/mL)
CON	0	4.19^##^	40.71^##^	3132^##^	3.45^##^	54.10^##^	3421^##^	4.80^##^	26.11^##^	3583
Inorganic Zn- CON	50	3.06	55.51	3075	1.96	62.29	4174	3.73	64.22	3425
Inorganic Zn^$^	30 ^&^	3.23	72.21^a^	2726^bc^	2.99^bcd^	26.87^c^	3362	3.94^ab^	85.67^a^	2935
	60 ^&^	2.49	64.60^a^	2221^c^	4.47^a^	75.48^b^	3664	4.50^a^	70.43^ab^	3741
	90 ^&^	2.60	37.20^b^	3106^b^	3.41^bc^	60.90^b^	2890	2.72^cd^	62.28^b^	3299
Organic Zn^@^	30 ^&^	2.31	68.09^a^	3789^a^	2.17^d^	94.31^a^	4089	2.04^d^	54.30^b^	3448
	60 ^&^	1.98	64.62^a^	3155^b^	2.89^cd^	67.88^b^	4205	3.23^bc^	84.61^a^	3722
	90 ^&^	2.70	61.63^a^	2917^b^	3.82^b^	69.51^b^	3520	3.87^ab^	81.90^a^	3512
SD		0.38	11.31	472.35	0.73	20.26	442.26	0.82	11.87	273.93
Zn source	Inorganic Zn	2.77^a^	58.00^b^	2684^b^	3.62^a^	54.43^b^	3305^b^	3.72^a^	72.83	3325
	Organic Zn	2.33^b^	64.78^a^	3287^a^	2.96^b^	77.19^a^	3938^a^	3.05^b^	73.56	3561
SD		0.22	3.39	301.50	0.33	11.40	316.50	0.34	0.40	118.00
Zn level	30	2.77^a^	70.15^a^	3258^a^	2.58^b^	60.60	3725^b^	2.99	70.0	3192^c^
	60	2.24^b^	64.61^b^	2688^c^	3.68^a^	71.71	3934^a^	3.87	77.5	3731^a^
	90	2.65^a^	49.42^c^	3012^b^	3.61^a^	65.20	3205^c^	3.30	72.1	3406^b^
SD		0.23	8.76	233.43	0.50	4.56	306.50	0.36	3.16	221.60
*P value*	Zn source	0.011	0.085	0.001	0.008	<0.001	0.003	0.028	0.870	0.106
	Zn level	0.033	<0.001	0.020	0.001	0.077	0.002	0.058	0.425	0.013
	Zn source* Zn level	0.052	0.008	0.004	0.006	<0.001	0.894	<0.001	<0.001	0.322

^1^All groups are subject to heat stress. Values represent the average of 1-8 replicate cages.

^a-d^ Values with different superscript letters in the same column differ significantly (P < 0.05).

^$^Inorganic Zn: ZnSO_4_.7H_2_O.

^@^Organic Zn: Medium chelation strength organic protein Zn.

^&^Zn is added to the positive control.

^#^It indicates that CON group is significantly different from the single degree-of-freedom comparison performed in groups 2-8 (P<0.05). ^##^Indicates a high significant difference (P<0.01).

## 4 Discussion

Growth performance serves as a dependable metric for evaluating the health and developmental status of animals. Zinc, an indispensable trace element in the animal diet, assumes a crucial role in animal growth and development. However, broiler chickens, known for their abbreviated growth cycle and rapid metabolism, exhibit an elevated demand for zinc compared to other animals. Various research studies have suggested that heat stress damages intestinal morphology, impacting the feed intake and daily weight gain of broiler chickens (37, 38). In our investigation, we observed that Zn supplementation significantly increased the feed-to-weight ratio broilers during the experimental period under heat stress in comparison to the control group fed a standard diet. Therefore, a tentative conclusion can be drawn that the addition of zinc to the diets positively influences the feed/weight gain ratio of broiler chickens in response to heat stress.

The villus height in the small intestine serves to maximize the absorptive surface area, facilitating efficient nutrient absorption and contributing to overall digestive function. Previous studies have documented that heat stress can lead to damage to the villi the apex of the small intestine in broiler chickens, significantly decreasing intestine villus height (39–41). In agree with previous studies, our study revealed that Xueshan chicken under heat stress exhibited a decrease VH, VD and VH/CD in the jejunum and ileum, while the zinc supplementation restored these. Notably, the organic zinc groups demonstrated a more pronounced effect, with values surpassing those of the inorganic zinc groups throughout the experimental period, which is consistent with one previous study (42). This suggests the potential role for zinc (especially organic zinc) in mitigating heat-stress-induced intestinal injury. Therefore, the addition of zinc supplementation could positively mitigate and improve the intestinal morphology.

Generally, there is a strong correlation among the risk of stress, intestinal morphology and oxidative stress. MDA is an end product of free-radical chain reaction and lipid peroxidation (43), hence, it is frequently used in the measurement of lipid peroxide levels and provides a good correlation with the degree of lipid peroxidation (44). In present study, heat stress increased the serum MDA content in the serum of chickens, however, zinc supplementation, especially organic zinc, decreased the serum MDA, which indicated that zinc ameliorated oxidative stress induced by heat stress in the chicken. Moreover, present study revealed that zinc supplementation improved SOD and GSH-px activities in the serum of chicken. SOD and GSH-px are known for their protective function in eliminating reactive free radicals, and thus it representing an important antioxidant defense in nearly all cells exposed to oxygen (45). Specifically, zinc is an essential component of SOD, which has an important function in the detoxification of superoxide free radicals and protection of cells against oxidative stress by catalyzing the conversion of superoxide anion (O_2_^−^) to H_2_O_2_. Cu-ZnSOD is the major superoxide scavenger in the cytoplasm, nucleus, lysosomes, and intermembrane space of mitochondria (46, 47). Several studies have reported the use of zinc enhances some activities of antioxidant enzymes includes SOD (48–51). Therefore, we also observed that zinc supplementation improved SOD and GSH-px activities in the serum of chickens, demonstrating that adding zinc into the diet is an effective strategy against heat stress-induced oxidative stress in chickens.

In conclusion, this study sheds light on the benefits of zinc supplementation in alleviating the adverse effects of heat stress on broiler chickens. It improves intestinal morphology and enhances antioxidant defense mechanisms, suggesting that zinc supplementation (especially organic zinc) may be an effective strategy to maintain poultry health and performance under heat-stress conditions. This study contributes valuable insights to the scientific and rational zinc supplementation in broiler chicken production under heat stress.

## Data availability statement

The original contributions presented in the study are included in the article/**Supplementary Material**. Further inquiries can be directed to the corresponding authors.

## Ethics statement

The animal research proposal was approved by the Institutional Animal Care and Utilization Committee (IACUC) of the Animal Experimental Ethics Committee of Yangzhou University (Permit Number: SYXK (SU) IACUC 2012-0029). The study was conducted in accordance with the local legislation and institutional requirements.

## Author contributions

PH: Writing – original draft, Data curation, Formal Analysis, Supervision. KL: Data curation, Formal Analysis, Software, Writing – original draft. XP: Data curation, Formal Analysis, Software, Writing – original draft. TY: Data curation, Formal Analysis, Software, Writing – original draft. CZ: Investigation, Writing – original draft. HG: Investigation, Resources, Writing – original draft. H-YL: Supervision, Writing – review & editing. M-AS: Software, Supervision, Writing – review & editing. YH: Data curation, Formal Analysis, Investigation, Writing – review & editing. WE: Conceptualization, Methodology, Supervision, Writing – original draft. XL: Conceptualization, Methodology, Supervision, Writing – original draft, Writing – review & editing. DC: Conceptualization, Project administration, Supervision, Validation, Writing – original draft, Writing – review & editing.

## References

[1] Sahin K Sahin N Kucuk O Hayirli A Prasad A. Role of dietary zinc in heat-stressed poultry: A review. Poultry Sci (2009) 88(10):2176–83. doi: 10.3382/ps.2008-00560 19762873

[2] Deshpande JD Joshi MM Giri PA. Zinc: The trace element of major importance in human nutrition and health. Int J Med Sci Public Health (2013) 2(1):1–6. doi: 10.5455/ijmsph.2013.2.1-6

[3] McClure S. How minerals may influence the development and expression of immunity to endoparasites in livestock. Parasite Immunol (2008) 30(2):89–100. doi: 10.1111/j.1365-3024.2007.00996.x 18186769

[4] Naz S Idris M Khalique M Zia-Ur-Rahman Alhidary I Abdelrahman M. The activity and use of zinc in poultry diets. World's Poultry Sci J (2016) 72(1):159–67. doi: 10.1017/S0043933915002755

[5] Farooq A Patoary MK Zhang M Mussana H Li M Naeem MA. Cellulose from sources to nanocellulose and an overview of synthesis and properties of nanocellulose/zinc oxide nanocomposite materials. Int J Biol macromolecules (2020) 154:1050–73. doi: 10.1016/j.ijbiomac.2020.03.163 32201207

[6] Abd El-Hack M Alagawany M Arif M Chaudhry M Emam M Patra A. Organic or inorganic zinc in poultry nutrition: a review. World's Poultry Sci J (2017) 73(4):904–15. doi: 10.1017/S0043933917000769

[7] Suttle NF. Mineral nutrition of livestock. 4th ed. Oxfordshire, OX, UK: Cabi (2010).

[8] Upadhayay U Vishwa PCV. Growth promoters and novel feed additives improving poultry production and health, bioactive principles and beneficial applications: the trends and advances-a review. Int J Pharmacol (2014) 10(3):129–59. doi: 10.3923/ijp.2014.129.159

[9] Shilja S Sejian V Bagath M Mech A David C Kurien E. Adaptive capability as indicated by behavioral and physiological responses, plasma HSP70 level, and PBMC HSP70 mRNA expression in Osmanabadi goats subjected to combined (heat and nutritional) stressors. Int J Biometeorology (2016) 60:1311–23. doi: 10.1007/s00484-015-1124-5 26698161

[10] Al-Dawood A. Towards heat stress management in small ruminants-a review. Ann Anim Sci (2017) 17(1):59. doi: 10.1515/aoas-2016-0068

[11] Nawab A Ibtisham F Li G Kieser B Wu J Liu W. Heat stress in poultry production: Mitigation strategies to overcome the future challenges facing the global poultry industry. J thermal Biol (2018) 78:131–9. doi: 10.1016/j.jtherbio.2018.08.010 30509629

[12] Etches R John T Gibbins AV. Behavioural, physiological, neuroendocrine and molecular responses to heat stress. Poultry production in hot climates. Wallingford UK: CABI (2008) p. 48–79.

[13] Zheng Y Xie T Li S Wang W Wang Y Cao Z. Effects of selenium as a dietary source on performance, inflammation, cell damage, and reproduction of livestock induced by heat stress: A review. Front Immunol (2022) 12:820853. doi: 10.3389/fimmu.2021.820853 35116042 PMC8803637

[14] Guarino Amato M Castellini C. Adaptability challenges for organic broiler chickens: A commentary. Animals (2022) 12(11):1354. doi: 10.3390/ani12111354 35681819 PMC9179304

[15] Lian P Braber S Garssen J Wichers HJ Folkerts G Fink-Gremmels J. Beyond heat stress: Intestinal integrity disruption and mechanism-based intervention strategies. Nutrients (2020) 12(3):734. doi: 10.3390/nu12030734 32168808 PMC7146479

[16] Daghir N. Nutritional strategies to reduce heat stress in broilers and broiler breeders. Lohmann Inf (2009) 44(1):6–15.

[17] Kucuk O Sahin N Sahin K. Supplemental zinc and vitamin A can alleviate negative effects of heat stress in broiler chickens. Biol Trace element Res (2003) 94:225–35. doi: 10.1385/BTER:94:3:225 12972690

[18] Yang G Chan PH Chen J Carlson E Chen SF Weinstein P. Human copper-zinc superoxide dismutase transgenic mice are highly resistant to reperfusion injury after focal cerebral ischemia. Stroke (1994) 25(1):165–70. doi: 10.1161/01.STR.25.1.165 8266365

[19] Liao X Li W Zhu Y Zhang L Lu L Lin X. Effects of environmental temperature and dietary zinc on egg production performance, egg quality and antioxidant status and expression of heat-shock proteins in tissues of broiler breeders. Br J Nutr (2018) 120(1):3–12. doi: 10.1017/S0007114518001368 29936928

[20] Kiarie E Walsh M Nyachoti C. Performance, digestive function, and mucosal responses to selected feed additives for pigs. J Anim Sci (2016) 94(suppl_3):169–80. doi: 10.2527/jas.2015-9835

[21] El-Katcha M Soltan MA El-Badry M. Effect of dietary replacement of inorganic zinc by organic or nanoparticles sources on growth performance, immune response and intestinal histopathology of broiler chicken. Alex J Vet Sci (2017) 55(2):129–45. doi: 10.5455/ajvs.266925

[22] Zhu C Lv H Chen Z Wang L Wu X Chen Z. Dietary zinc oxide modulates antioxidant capacity, small intestine development, and jejunal gene expression in weaned piglets. Biol Trace element Res (2017) 175:331–8. doi: 10.1007/s12011-016-0767-3 27339255

[23] Huang L Li X Wang W Yang L Zhu Y. The role of zinc in poultry breeder and hen nutrition: an update. Biol Trace Element Res (2019) 192:308–18. doi: 10.1007/s12011-019-1659-0 30767181

[24] Halliwell B. Role of free radicals in the neurodegenerative diseases: therapeutic implications for antioxidant treatment. Drugs Aging (2001) 18:685–716. doi: 10.2165/00002512-200118090-00004 11599635

[25] Wu D Cederbaum AI. Alcohol, oxidative stress, and free radical damage. Alcohol Res Health (2003) 27(4):277. 15540798 PMC6668865

[26] Lin H Jiao H Buyse J Decuypere E. Strategies for preventing heat stress in poultry. World's Poultry Sci J (2006) 62(1):71–86. doi: 10.1079/WPS200585

[27] Saleh AA Ragab MM Ahmed EA Abudabos AM Ebeid TA. Effect of dietary zinc-methionine supplementation on growth performance, nutrient utilization, antioxidative properties and immune response in broiler chickens under high ambient temperature. J Appl Anim Res (2018) 46(1):820–7. doi: 10.1080/09712119.2017.1407768

[28] Wasti S Sah N Mishra B. Impact of heat stress on poultry health and performances, and potential mitigation strategies. Animals (2020) 10(8):1266. doi: 10.3390/ani10081266 32722335 PMC7460371

[29] De Grande A Leleu S Delezie E Rapp C De Smet S Goossens E. Dietary zinc source impacts intestinal morphology and oxidative stress in young broilers. Poultry Sci (2020) 99(1):441–53. doi: 10.3382/ps/pez525 PMC7587869 32416829

[30] Abuajamieh M Abdelqader A Irshaid R Hayajneh FM Al-Khaza'leh J Al-Fataftah A-R. Effects of organic zinc on the performance and gut integrity of broilers under heat stress conditions. Arch Anim Breed (2020) 63(1):125–35. doi: 10.5194/aab-63-125-2020 PMC7201270 32382654

[31] Jiang ZY LX Jiang SQ Zhou GL Lin YC Chen F Ma XY. Zinc requirement of yellow broilers from forty-three to sixty-three days of age. J Scientia Agricultura Sinica (2010) 43:4295–302.

[32] Takashima M Yaguchi J. High-solids thermophilic anaerobic digestion of sewage sludge: effect of ammonia concentration. J Material Cycles Waste Manage (2021) 23:205–13. doi: 10.1007/s10163-020-01117-z

[33] Ullah S Mustafa S Ennab W Muhammad J Shafiq M Kavita NM. A protective role of resveratrol against the effects of immobilization stress in corpora lutea of mice in early pregnancy. J Integr Agric (2020) 19(7):1857–66. doi: 10.1016/S2095-3119(19)62856-X

[34] Ennab W Mustafa S Wei Q Lv Z Kavita NM Ullah S. Resveratrol protects against restraint stress effects on stomach and spleen in adult male mice. Animals (2019) 9(10):736. doi: 10.3390/ani9100736 31569722 PMC6826970

[35] Ennab W Ye N Wu H Ullah S Hadi T Bassey AP. The synergistic effects of the combination of L-carnitine and lycopene on the lycopene bioavailability and duodenal health of roosters. Animals (2023) 13(8):1274. doi: 10.3390/ani13081274 37106837 PMC10134981

[36] Lee K Shin J. Equivalent single-degree-of-freedom analysis for blast-resistant design. Int J Steel Structures (2016) 16:1263–71. doi: 10.1007/s13296-016-0073-0

[37] Quinteiro-Filho WM Gomes A Pinheiro ML Ribeiro A Ferraz-de-Paula V Astolfi-Ferreira CS. Heat stress impairs performance and induces intestinal inflammation in broiler chickens infected with Salmonella Enteritidis. Avian Pathol (2012) 41(5):421–7. doi: 10.1080/03079457.2012.709315 22900578

[38] Sandner G Mueller AS Zhou X Stadlbauer V Schwarzinger B Schwarzinger C. Ginseng extract ameliorates the negative physiological effects of heat stress by supporting heat shock response and improving intestinal barrier integrity: Evidence from studies with heat-stressed Caco-2 cells, C. elegans growing broilers Molecules (2020) 25(4):835. doi: 10.3390/molecules25040835 32075045 PMC7070719

[39] Liu G Zhu H Ma T Yan Z Zhang Y Geng Y. Effect of chronic cyclic heat stress on the intestinal morphology, oxidative status and cecal bacterial communities in broilers. J Thermal Biol (2020) 91:102619. doi: 10.1016/j.jtherbio.2020.102619 32716869

[40] Nanto-Hara F Kikusato M Ohwada S Toyomizu M. Heat stress directly affects intestinal integrity in broiler chickens. J Poultry Sci (2020) 57(4):284–90. doi: 10.2141/jpsa.0190004 PMC7596036 33132728

[41] Santos RR Awati A Roubos-van den Hil PJ Tersteeg-Zijderveld MH Koolmees PA Fink-Gremmels J. Quantitative histo-morphometric analysis of heat-stress-related damage in the small intestines of broiler chickens. Avian Pathol (2015) 44(1):19–22. doi: 10.1080/03079457.2014.988122 25410755

[42] Chang Y Wang K Wen M Wu B Liu G Zhao H. Organic zinc glycine chelate is better than inorganic zinc in improving growth performance of cherry valley ducks by regulating intestinal morphology, barrier function, and the gut microbiome. J Anim Sci (2023) 101:skad279. doi: 10.1093/jas/skad279 37606553 PMC10494877

[43] Liu Y Han J Huang J Wang X Wang F Wang J. Dietary L-arginine supplementation improves intestinal function in weaned pigs after an Escherichia coli lipopolysaccharide challenge. Asian-Australasian J Anim Sci (2009) 22(12):1667–75. doi: 10.5713/ajas.2009.90100

[44] Cetinkaya A Bulbuloglu E Kantarceken B Ciralik H Kurutas EB Buyukbese MA. Effects of L-carnitine on oxidant/antioxidant status in acetic acid-induced colitis. Digestive Dis Sci (2006) 51(3):488–94. doi: 10.1007/s10620-006-3160-9 16614957

[45] Cheung C Zheng G Li A Richardson B Lam P. Relationships between tissue concentrations of polycyclic aromatic hydrocarbons and antioxidative responses of marine mussels, Perna viridis. Aquat Toxicol (2001) 52(3-4):189–203. doi: 10.1016/S0166-445X(00)00145-4 11239681

[46] Elchuri S Oberley TD Qi W Eisenstein RS Jackson Roberts L Van Remmen H. CuZnSOD deficiency leads to persistent and widespread oxidative damage and hepatocarcinogenesis later in life. Oncogene (2005) 24(3):367–80. doi: 10.1038/sj.onc.1208207 15531919

[47] Fukai T Ushio-Fukai M. Superoxide dismutases: role in redox signaling, vascular function, and diseases. Antioxidants Redox Signaling (2011) 15(6):1583–606. doi: 10.1089/ars.2011.3999 PMC3151424 21473702

[48] Aksu DS Aksu T Baytok E. The effects of replacing inorganic with a lower level of organically complexed minerals (Cu, Zn and Mn) in broiler diets on lipid peroxidation and antioxidant defense systems. Asian-Australasian J Anim Sci (2010) 23(8):1066–72. doi: 10.5713/ajas.2010.90534

[49] Chang Y Tang H Zhang Z Yang T Wu B Zhao H. Zinc methionine improves the growth performance of meat ducks by enhancing the antioxidant capacity and intestinal barrier function. Front Veterinary Sci (2022) 9:774160. doi: 10.3389/fvets.2022.774160 PMC8841862 35174244

[50] El-Bahr SM Shousha S Albokhadaim I Shehab A Khattab W Ahmed-Farid O. Impact of dietary zinc oxide nanoparticles on selected serum biomarkers, lipid peroxidation and tissue gene expression of antioxidant enzymes and cytokines in Japanese quail. BMC veterinary Res (2020) 16(1):1–12. doi: 10.1186/s12917-020-02482-5 PMC7510065 32967666

[51] Georgieva NV Gabrashanska M Koinarski V Yaneva Z. Zinc supplementation against Eimeria acervulina-induced oxidative damage in broiler chickens. Vet Med Int (2011) 2011:647124. doi: 10.4061/2011/647124 21461390 PMC3065002

