# Supplementary material for: Zinc intake ameliorates intestinal morphology and oxidative stress of broiler chickens under heat stress
Source: Front Immunol. 2024 Jan 8;14:1308907. doi: 10.3389/fimmu.2023.1308907 (PMC10800777; doi:10.3389/fimmu.2023.1308907)
Supplement: Supplementary file 1 [file DataSheet_1.docx]

**Table S1. Composition and nutrient levels of the basal diets for 60-102 broilers (as-fed basis)**

| **Item** | **Basal diet** |
| --- | --- |
| Ingredient（%） |  |
| Corn | 76.27 |
| Soybean meal | 19.50 |
| Soybean oil | 1.38 |
| DL-Met | 0.12 |
| L-Lys | 0.13 |
| CaHPO_4_⋅2H_2_O | 0.79 |
| CaCO_3_ | 1.15 |
| NaCl | 0.30 |
| Micronutrients^1^ | 0.26 |
| Cornstarch + Zn^2^ | 0.10 |
| Nutrient levels composition |  |
| ME, Kcal/kg | 3037 |
| CP^4^, % | 15.31 |
| Lys, % | 0.81 |
| Met, % | 0.36 |
| L- Thr, % | 0.57 |
| Try, % | 0.16 |
| Met+cys, % | 0.60 |
| Ca, % | 0.69 |
| P, % | 0.45 |
| Nonphytate P, % | 0.22 |
| Zn^4^, mg/kg | 18.33 |

^1^ VA 6000 IU, VD3 2250 IU, VE 16.5 IU, VK3 1.5 mg, VB1 1.5 mg, VB2 4.8 mg. VB6 2.25 mg, VB12 0.015 mg. Pantothenic acid calcium 7.5 mg, Niacin 27 mg, Folic acid 0.75 mg, Biotin 0.075 mg, Choline 750 mg, Cu (CuSO4·5H2O) 7 mg, Fe (FeSO_4_·7H_2_O) 40 mg, Zn (ZnSO4·7H2O) 0mg, Mn (MnSO4.H2O) 40 mg, Se (Na2SeO3) 0.15 mg, I (Ca(IO_3_)_2_·H2O) 0.5 mg.

^2^ Zn supplements added in place of equivalent weights of cornstarch.

**Table S2. Effects of different Zn sources and Zn levels on Growth Performance and mortality of Jiangsu Xueshan broilers aged 61-102 days under heat stress^1^**

| **Items** | **Zn level(mg/kg)** | **ADG (g/d)** | **ADF (g/d)** | **Feed-to-gain ratio** | **Mortality (%)** |  |
| --- | --- | --- | --- | --- | --- | --- |
|  |  |  |  |  |  |  |
| CON | 0 | 15.7^#^ | 65.3 | 4.21^##^ | 0 |  |
| Inorganic Zn-CON | 50 | 14.1 | 67.1 | 4.81 | 0 |  |
| Inorganic Zn^$^ | 30 ^&^ | 14.6 | 64.5 | 4.62 | 0 |  |
|  | 60 ^&^ | 14.9 | 67.6 | 4.67 | 0 |  |
|  | 90 ^&^ | 16.1 | 66.5 | 4.24 | 3.13 |  |
| Organic Zn^@^ | 30 ^&^ | 15.2 | 64.1 | 4.32 | 0 |  |
|  | 60 ^&^ | 13.8 | 64.5 | 4.98 | 3.13 |  |
|  | 90 ^&^ | 16.4 | 69.7 | 4.24 | 3.13 |  |
| SD |  | 0.88 | 2.02 | 0.27 | 1.57 |  |
| Zn source | Inorganic Zn | 15.2 | 66.2 | 4.50 | 0.78 |  |
|  | Organic Zn | 15.1 | 66.1 | 4.51 | 2.09 |  |
| SD |  | 0.05 | 0.05 | 0.01 | 0.66 |  |
| Zn level | 30 | 14.9 | 64.3 | 4.47 | 0 |  |
|  | 60 | 14.3 | 66.1 | 4.83 | 1.57 |  |
|  | 90 | 16.2 | 68.1 | 4.22 | 3.13 |  |
| SD |  | 0.79 | 1.55 | 0.25 | 1.28 |  |
| *P value* | Zn source | 0.992 | 0.977 | 0.946 | \ |  |
|  | Zn level | 0.173 | 0.340 | 0.248 | \ |  |
|  | Zn source*Zn level | 0.688 | 0.508 | 0.727 | \ |  |

**^1^**All groups are subjected to heat stress. Values represent the average of 1-8 replicate cages

^$^Inorganic Zn: ZnSO4.7H2O.

^@^Organic Zn: Medium chelation strength organic protein Zn.

^&^Zn is added to the positive control diet.

^#^It indicates that CON group is significantly different from the single degree-of-freedom comparison performed in groups 2-8 (*P*<0.05). ^##^Indicates a high significant difference (*P*<0.01).
